
JOURNAL INFORMATION
==============================
NLM Title Abbreviation: Med Chir Rev
NLM Journal ID: 0034617

The Medico-Chirurgical Review

Publisher: Biomedical Journal Digitization Project

ARTICLE INFORMATION
==============================
PMCID: PMC4966256
PMID: 29917627
Article version: 1
Subjects: Articles

Hospital Practice

Print publication date: 1828 Oct 1
Volume: 9
Issue: 18
First page: 553
Last page: 575
1828 Sep
License: This work is free of known copyright restrictions. For more information, please see PMC Back Issue Digitization.
License URL: https://creativecommons.org/publicdomain/mark/1.0/

==============================

### HOSPITAL PRACTICE.

### 75. HOTEL DIEU.

### I. Severe Burn followed by Tetanus.

There are no two diseases in all the black catalogue of human ills, more dreadful to endure, more horrible to witness, and we may add, more difficult to cure, than tetanus and hydrophobia. It is a singular fact in therapeutics, that the number of remedies for a given disease, is always in proportion to its *irremediability*. There is only one nostrum for the itch, because any one can cure it by brimstone, whilst innumerable specifics exist for hydrophobia, because it is curable by nobody! It would require even more than the "ora centum, ferrea vox" to enumerate the various modes of treatment in tetanus, many of which have been successful in the hands of their *proposers*, few or none in the trials of others. Cold affusion, warm affusion, mercury, bleeding, and laudanum, each and all, like the creeds of other times, have had their day, but speedily sunk into night—ad orcum sunt demissi! It is scarcely a twelve-month ago, since a letter was received from a surgeon in the navy, by one of the most eminent men in the profession, recommending the employment of sulphur held in the hand of the patient, as almost a specific for tetanus, and detailing some cases in which it had perfectly succeeded! It happened that the gentleman to whom the letter was addressed had a patient with tetanus in the hospital at the time, and determined to put the *specific* to the test. We need scarcely say that it did not a particle of good.

The only hope we possess, and, alas! it is a feeble one, of arriving at a rational treatment of tetanus, is by carefully studying its pathology. In very many cases there is nothing which the eye of the anatomist can at present detect as disease; but in others, congestion and inflammation are observed in the membranes investing the spinal marrow, and even in the medulla itself. In some cases, indeed, the disease would appear to be *spinitis*, or, it may be, to end in it. In the present imperfect and unsatisfactory state of our knowledge on the subject, the only thing that can be done is to place upon record the facts that occur, more as beacons to shew what has *not* been of use, than examples to indicate what *has*.

Jean Baptise Roger, ætatis 23, a journeyman, was received into the Hôtel Dieu, on the 11th of March, on account of an extensive burn, received whilst intoxicated, by his clothes having caught fire. The burn extended from the posterior part of the body, over the thighs, to the ham; the skin, for the most part, was converted into a hard, black eschar, around which was a zone where the integuments were not so much destroyed; the pulse was pretty strong; but the surface was cold. A bleeding was employed and emollient poultices applied upon the burn, but on the 13th, the reaction was considerable. *Bath—anodynes—poultices, with laudanum.*

On the 15th, the slough was beginning to be detached, and granulations were noticed springing up. The parts in the neighbourhood were the seat of inflammation, and the pain was extremely severe. On the 19th the patient complained of some pain in the head, which was greatly increased on the 20th. On this day there was noticed a difficulty in the motions of the neck, and stiffness in the right arm, together with a slight degree of trismus. The belly was painful upon pressure—the surface was hot—the pulse hard and frequent. The treatment consisted in a bleeding from the arm, leeches behind the ears, and an anodyne with laudanum in the evening, whilst the greatest precaution was observed in protecting the wound from the influence of the air. On the next day, the trismus was increased, and the muscles of the left arm and neck were affected; the face was bathed in sweat; the pulse very sharp. *Bleeding to syncope—bath for three hours—laudanum both by the mouth and in an enema—anodyne fomentations to the masseters and muscles of the neck.* On the 22nd, opisthotonos was established, and on the 23rd, the jaws were firmly locked. The suppuration from the wound had diminished—the granulations had a scarlet hue, the pain was excessive, the pulse became feeble, the surface was covered with a cold clammy sweat, and the patient expired at seven in the evening of the 23rd. The anodyne fomentations, &c. were continued on the last two days, and leeches applied to the back of the neck.

*Dissection* 36 *hours after death.* Those muscles of the body which had *not* been affected with tetanus, were firmly contracted, whilst those on the contrary which *had*, were completely relaxed. The whole body smelt strongly of laudanum. The pia mater was injected, and its veins much congested; the tunica arachnoides was opaque, and both membranes were with difficulty detached from the cortical substance of the brain, which was uniformly red and injected. The medullary substance near the surface was also extremely vascular, but as the ventricles were approached, the vascularity decreased, and indeed disappeared. The ventricles contained a very small quantity of serum; the corpora striata, and thalami nervorum opticorum had a number of blood-vessels ramifying on their surface, but were comparatively free from vascularity in their centre, and lastly the brain altogether was firmer than natural.

On opening the vertebral canal, the veins of the theca were found to be gorged with blood; the medulla itself was of natural consistence, but the grey matter in its centre was uniformly and remarkably rosy and injected, especially a little below the cervical portion, and again at the 8th or 9th vertebra of the back.

The great end of the stomach was a little reddened, and the same appearance was observed in the ileum and parts of the colon.

In a case of the disease which occurred very lately at St. George's Hospital, the sulphate of quinine was given for the sake of experiment. We shall mention the heads of the case.

II. WOUND IN THE SOLE OF THE FOOT, SUCCEEDED BY TETANUS—CURIOUS CONDITION OF THE INTERNAL PLANTAR NERVE.

A healthy-looking lad of fifteen, was climbing over the iron railings in the Park, when unluckily he slipped, and the spike pierced the sole of his right foot. This was on the 14th of July, and he felt for a day or two some pain in the foot and the calf of the leg, but it gradually subsided. On the 21st, he got wet and remained for some time without changing his clothes; on the morning of the 25th, he went out with his brother, but complained of some cramps in his belly and chest, which "were drawn into lumps," and was shortly obliged to return to the house. There was stiffness of the jaw, and there seems at this time to have been a degree of emprosthotonos. Towards the close of the day the symptoms grew severe, the jaw became locked in the night, which was spent in pain and misery, and on the morning of the 16th, the muscles of the back were affected. The wound, as has been mentioned, had been painful for a day or two, but under the use of a poultice a little suppuration took place, and it gave him no more inconvenience.

When brought to the hospital on the 16th, at 11, A. M. opisthotonos was completely established, the body being supported, when supine, on the shoulders and the heels—the jaw was nearly closed—the spasms very frequent and violent—the face bathed in sweat—the pulse full and quick.

Mr. Brodie arrived in the course of an hour, and ordered the patient to be placed on a bed-stead in the yard, and buckets of cold water to be emptied upon him. The affusion appearing to have rather an injurious effect, was given up, and the boy carried back to the ward. Towards the middle of the day he was placed in the cold bath at his own request, and experienced a trifling relief. The case being hopeless, Mr. Brodie determined on making an experiment with the sulphate of quinine, which was given at five in the afternoon, to the amount of 5 grains, and exhibited every hour in three-grain doses, as well as the difficulty of swallowing would allow;* at 7 P. M. the cold bath was repeated, and at 8, he took a drop of the hydrocyanic acid, with directions to repeat it every third hour. He had taken three drops, and at six in the morning of the 17th, was offered the fourth, but was totally unable to swallow it. The symptoms had been hitherto gradually increasing in severity, unchecked in the slightest degree by the means that were

* He took 59 grains in all.

employed, but now they assumed a peculiar and ominous character. The paroxysms of spasm were no longer confined to the voluntary muscles, but seized upon the diaphragm, the muscles of the larynx, and the heart. When the periodical convulsion came on he turned livid in the face, gave a long spasmodic gasp, like an animal that is strangled, the heart beat with violence for an instant, fluttered, and was still—the pulse grew weaker and weaker, and in one of these attacks he expired at 11, A. M.

Before we proceed to the dissection we may mention the appearance and condition of the wound. It was situated on the inside of the sole of the foot, apparently in the direction of the internal plantar nerve. On the patient's admission it was very nearly healed, presented no unhealthy appearance whatever, and the parts in its vicinity for about the dimension of a sixpence, were hardened and agglutinated together.

*Sectio cadaveris.* The membranes of the brain were injected, and the brain altogether decidedly more vascular than it should be ; there was also more serum than usual in the ventricles. The spine was laid open from occiput to sacrum, and on lifting the series of vertebral arches, a peculiar appearance was noticed on the outside of the theca. A transparent sort of substance looking like very fine adeps, or cellular membrane injected with serum was collected in considerable quantity. It had not the appearance of lymph, and appeared to be a natural formation.

The principal interest attached to the examination of the wound. The posterior tibial nerve in the leg was found to be perfectly healthy, but on tracing that branch of its plantar division that goes to supply the great toe, it was found to be involved in the wound. The spike had not actually penetrated the nerve, but passed by the side of it, and probably grazed it, so that it lay in the centre of the lymph and induration produced by the injury.

In his clinical lecture Mr. Brodie alluded to a similar case that occurred to the late Mr. Ewbank. A man who had been stabbed with a pitchfork in the leg, became affected with tetanus and died. On dissection, the fork was discovered to have penetrated to the peroneal nerve, which appeared to have been bruised, and was involved, as in this case, in the inflammation excited in the wound. In cases like these, it would not be unreasonable to propose the excision of the cicatrix, because if unattended with good, it could scarcely be followed by harm, and would be to the surgeon what straws are to men that are drowning—bad support certainly, but probably the best they can get. That tetanus (traumatic) will follow other injuries than those of the nerves, at least the larger ones, we are aware, indeed the case at the head of this article, where the cause was a burn, is sufficient to settle the question. At the same time it is known, that an injury of a part which is largely supplied with the branches of nerves will, cæteris paribus, be infinitely more liable to be followed by tetanus, than the wound of a part which is not so supplied. Hence we account for the comparative frequency of the disease after injuries of the hand, or the sole of the foot, where the nerves are both numerous and large. Those anomalous symptoms which so frequently follow the lesion of a nerve, would seem to be similar in *kind*, though widely remote in *degree*, from the horrible spasm in tetanus, which seems to affect every muscle in the frame. The case of the medical gentleman, which we published, is a melancholy instance of this very distressing affection, and another of a similar description was detailed by Mr. Bell, in his lectures, we believe, at the College.

A man, while at work on the side of a vessel in dock, slipped from the platform and fell, but was caught in his descent, and hung suspended by the ham from a boat which was slung to the ship. Contusion and inflammation of the part were the consequence, but these in a little while subsided. The immediate effects of the injury being over, there slowly and gradually came on the most excruciating pain in the sole of the foot, so unbearable at times, that the poor man would frequently rise from his bed to plunge the leg in the hottest water, and suddenly remove it to the hearth-stone, or place it in ice if he could get it. He went to a hospital, where he took a course of mercury, but experienced no relief and was dismissed. The pains becoming worse, he was re-admitted, but only to be treated with the same ill success. He gradually sank under the influence of pain, and it was only a few days previous to his death that the patient was seen by Mr. Bell. On examination, Mr. B. found a little knotty tumour in the ham, which was exquisitely painful on pressure, and appeared to be seated in the course of the nerve. It was too late to perform an operation, or else it was proposed, if the state of the patient had allowed it, to cut across the nerve. On dissection, the nerve (we think the posterior tibial) was found to be expanded in the ham into a sort of ganglionic knot, which embraced and divided its filaments, and had caused all the sufferings in the sole of the foot.

This case has a certain analogy with those which preceded, and we know of no reason why the spasms of tetanus should not be as likely to depend on a lesion of a nerve, as all these anomalous and generally indomitable symptoms. In a case which is mentioned in Sir Astley Cooper's Lectures, "the whole sciatic nerve was included in a ligature, which was applied to suppress hæmorrhage from the artery which accompanies the nerve. In four days the man was seized with violent spasm in the stump. On the fifth day spasms affected the limb, and from thence extended to the other muscles of the body. On the seventh day he died."

If the above was not allied to tetanus, or tetanus itself, we are very much mistaken. Mr. Brodie, in alluding to excision of the wound, made a very important remark. Allowing, said Mr. B. that the general spasms are the consequences of the local affection, still there is neither probability nor analogy in favour of the notion that the former will subside if the latter is removed. The wound is the cause, it is true; but when once the constitution is affected, this affection becomes independent of its cause, and will not be removed by its removal. Look, then, which way we will, we see but how little we know of the laws of the nervous system, either in health or disease. Whether the progress of knowledge and "march of mind" will further discover our weakness or augment our strength, if strength it can be called, is a problem which we leave for those to solve who can.

---

### 76. ST. BARTHOLOMEW'S HOSPITAL.

#### VARICOSE VEINS.

We had thought that the minds of the profession in this country were pretty well made up against tying or cutting out portions of varicose veins. Sir Astley Cooper, whose experience, of course, has been great, has publicly abandoned the operation, or rather operations, and the example, we know, has been followed by surgeons of very great eminence. A man may go on with success for a time, but at last the day of reckoning arrives, and the loss of his patient convinces him, alas! when too late, of the dangers and disasters attending the practice.

One of these "warnings" has lately occurred at St. Bartholomew's Hospital, and we give the particulars that those persons may profit by it who are able or willing to profit by any thing, for the obstinate routinist is neither.

*Case.* J. D. ætatis 35, received, when a boy, a scald on the inside of the leg, where there formed in after years a varicose vein, and an ulcer below the internal malleolus. The occasional bursting of the vein, and pain and inconvenience of an ulcer, preventing him from following his business as a servant, he entered Bartholomew's Hospital on the 26th of May. His habit of body was bilious and irritable—his bowels were costive—his pulse rather quick, but variable and full. The treatment, in the first instance, consisted in the application of poultices and leeches, attention to the diet, and rest in bed. In the course of a fortnight strapping was employed, but discontinued at the end of a week, having only been productive of pain and inconvenience. Emollients were therefore resumed, no amendment took place, and, on the 25th of June, "the vein was exposed about the middle of the leg, and a third of an inch was removed with a very slight hæmorrhage." He bore the operation very ill, and immediately afterwards complained of a sense of oppression and sinking at his chest. Twenty drops of laudanum were given, the limb kept continually wetted with cold lotion, and the bowels not having been opened in the day, he took three grains of calomel, combined with ten of jalap.

On the following morning the bowels were unopened, there was nausea and vomiting of greenish-coloured matter, and the absorbents of the thigh were swollen, reddened, and painful to the touch. The calomel and jalap were repeated, with a rhubarb draught, as salines produced sickness, and the addition at 2 P.M. of twenty leeches to the thigh, and two grains of blue pill, with half a grain of tartarized antimony. The bowels were relieved, and the pain of the limb was diminished, but the symptoms on the 27th indicated plainly a high degree of inflammation of the veins and absorbents. The dressings were removed, when the wound was found open, and its edges inflamed. Thirty-six leeches to the thigh—salines with antimony. V. S. ad ℥xx.—acid drinks—poultice—low diet.

The pulse before the bleeding was 120, but afterwards rose to 135 or 140. The patient passed a restless night, and the 28th found the leg and foot erysipelatous and swollen—epigastrium painful—sickness and malaise. To these symptoms there was added, what is always to be looked on as formidable in cases of phlebitis, a difficulty of breathing. The leeches were repeated with blue pill and opium, and the next day exhibited a pause in the severity of the symptoms. On the 30th, the first of a series of curious phenomena appeared, viz. the left arm was attacked with a phlegmonous inflammation. Lotion was applied, but the dyspnœa and malaise were so much increased in the night, that the house-surgeon ordered ten grains of the pulvis doveri.

*July 1st.* The inflammation and swelling in the leg have subsided, but continue in the arm, and have also appeared in the opposite leg, or, we should say, increased, for they appeared the day before. The pulse is improved, but still he complains of dyspnœa and pain in epigastrio. *Pulv. ip. comp.* grs. v. *ter die.* The leech-bites now ulcerated, and hæmorrhage took place from one of them to nearly a pint. Pain and tension were complained of in the head, depression of the powers supervened, and those peculiar symptoms of inflammation of the veins which all who have seen will readily recognize, carried off the patient in the evening of the 4th.

The cornea of both eyes, on the 3d, became opaque, the vessels of the tunica conjunctiva were loaded, the patient lost his sight, and was unable to open his eyes without a considerable effort. On the afternoon of the 3d, it was suggested that mercury had been useful "in cases attended with similar typhoid symptoms." The patient was accordingly ordered two grains of the submuriate and half a grain of opium every fourth hour. The effect, we need scarcely say, was—none; indeed the person or the drug that could do any good in the last stage (the typhoid) of phlebitis, would be worth ten times more than the fine of the judges in Athens, their own weight in gold, to the Patron of Physic, the Pythian Apollo!

*Dissection.* The vein which was a branch of the saphena minor was inflamed in the whole of its extent to the ham, where the inflammatory appearances ended abruptly. The vessel was filled with lymph and pus, several of its muscular branches were also filled with pus, and the inflammation extended for three quarters of an inch below the point where the operation was performed. Deep-seated abscesses had formed beneath the fascia of the left leg and fore-arm, separating to a considerable extent the fibres of the muscles; in the right fore-arm also a good deal of sero-purulent effusion was found in the same situation. There was nothing observed in the abdomen, but an abscess was discovered in the upper part of the lung on the right side, that was evidently the product of recent inflammation. There was a good deal of effusion into the cellular tissue of the pia mater, particularly towards the basis, "and the serum in the *veins* (qy. ventricles?) was of a deep yellow colour. Lymph was effused around the trunk of the carotids, the 3d nerve on the left side was evidently flattened and softer than its fellow, and the 5th on the right side had undergone a similar change to a greater extent. The corneæ of both eyes were rough and opaque, the crystalline of the right so softened as to yield to the slightest touch, the vitreous humour of a reddish yellow colour, and injected with red vessels, and the retina deeply reddened also.—*Gazette.*

We do not see at all why simple division of the vein with a valvular opening in the skin should not have been resorted to. We never saw an instance where this operation was followed by any bad effects, though of course their occurrence is by no means impossible. Nothing can be more unattended with danger, in general, than phlebotomy, and yet it is occasionally followed by fatal inflammation, a circumstance which should put us on our guard against blindly confiding in the absolute safety of any operation on the veins. Our only alternative in evils like these is merely to choose the least, and we think there can be but one opinion as to which is most likely to cause inflammation—division, or excision of a varicose vein.

So much for the operation, but a word or two remains to be said on the points of the case. In the first place we may remark that the patient was of an anxious, irritable habit, had been a servant, and probably led an indolent luxurious life, all of these circumstances exceedingly likely to favour the development of unhealthy inflammation. The different abscesses which were formed in each of both the lower and upper extremities, and in the lung, are exceedingly curious phenomena, though generally occurring in phlebitis in a greater or lesser degree. The case which is detailed by Mr. Lawrence in the sequel to Mr. Rose's paper in the Medico-Chirurgical Transactions is very analogous to the present, though the various formations of matter in that were a consequence of the operation of lithotomy. To us it appears that these purulent depôts in so many and various situations are very strong arguments in favour of those who conceive that a general vitiation of the fluids takes place; at any rate, we know of no other explanation, that has any pretence to feasibility. The changes which occurred in the eyes are difficult to account for. The reporter of the case puts the question—"Were they connected with the alterations which had taken place in the structure of the third and fifth pair of nerves?" The thing is more easily asked than answered.

---

### 77. LA CHARITE.

### STRANGULATED FEMORAL HERNIA. SLOUGHING OF THE INTESTINE—DEATH.

A woman of seventy had been affected for several years with reducible femoral hernia on both sides, that on the right being the oldest and largest. She had long worn a bandage, but having left it off for some time, the hernia on the left side became irreducible, and vomiting at first of bilious, afterwards of stercoraceous matters, together with costiveness succeeded. On the 16th June, six days after the hernia became irreducible, she entered La Charité, and was placed under the care of M. Roux. The vomiting was continual; the tumour about the size of a large walnut, painful and tender, especially on pressure; the integument which covered it hot and inflamed. The general symptoms were not so alarming. The abdomen was not very painful, the surface preserved its ordinary temperature, the pulse was pretty strong, and there was none of that cold clammy sweat which indicates the sloughing of strangled intestine. The operation, however, was immediately performed. The sac was exposed by a longitudinal incision, and the hernia, which consisted entirely of a small knuckle of intestine, was laid bare. Gimbernat's ligament was divided when there flowed out a yellowish fluid, evidently fæcal. The intestine was, therefore, drawn down to discover its state, and a cut was discovered in it corresponding to the edge of Gimbernat's ligament. The gut was dark brown, and inflamed both above and below the point of strangulation. An incision was made along the whole of its extent, and the fæces allowed to escape from the gut which was not fixed in the wound by any artificial means whatever.

The vomiting ceased on the completion of the operation, and on the 17th, the abdomen though a little tympanitic was not at all tender on pressure. A female catheter was introduced into the upper end of the intestine and vent given to gas and fæcal matter. On the 18th the flatulence was greater, but the state of the patient on the whole was satisfactory. The good symptoms, however, disappeared next day; the pulse grew weaker, the extremities cold, the belly though still free from pain was greatly distended, and the patient expired in the course of the 24th.

*Dissection.* There were every where marks of acute peritonitis, and adhesions between various folds of intestine, some of them apparently of ancient date. The large intestine was thickened in part of its extent, and its mucous membrane presented a deep brown hue. The portion of intestine which had formed the hernia had contracted slight adhesions with the walls of the abdomen, in the neighbourhood of the femoral ring. All that part of the intestine above the strangulation was distended with liquid fæcal matters and gas; the stomach presented the hour-glass contraction, and contained in its cavity a small quantity of the matters found in the intestine. Gimbernat's ligament, which had been fairly divided by the bistoury, had cut through the intestine like a fine and sharp ligature.

The reporter observes that three cases of a similar description have successively occurred at the Hospital of La Charité. This division of the gut by the stricture of Gimbernat's ligament is infinitely more likely to occur where intestine alone is included in the sac than when it is encircled by yielding omentum.

---

### 78. ROYAL INFIRMARY OF EDINBURGH.*

Dr. Ballingall deserves, and we trust will obtain, the approbation of the students of the Royal Infirmary for his clinical instruction and clinical reports. To descant on the merits of publishing reports of the practice of Hospitals, would at present be a work of complete supererogation, and save and except a few bigoted imbeciles, there is scarcely a practitioner from John o'Groat's House to the Lizard's Point, who does not acknowledge their power and value. Dr. Ballingall has furnished a useful and honourable example to his brethren, in recording the cases confided to his care, an example, that is like to be a good deal more praised than pursued. We wish it were possible, but fear it is not, to induce the provincial surgeons and physicians to imitate the plan of Dr. Ballingall, and give to the world the more interesting cases occurring, as they constantly must do, in the county and country hospitals. In London itself, the existence of regular hospital-reporters, prevents the necessity for the medical officers publishing or writing in propriâ personâ.

Dr. B. begins his lecture by referring to and concluding some cases in his last. These we pass over, and proceed to the case of William Gardener, aged 19, admitted with a lacerated wound of the hand, in consequence of the bursting of a fowling piece.

"The metacarpal bones of the left hand were fractured near their carpal extremities, their heads, with the exception of that of the thumb, were dislocated at their articulation with the bones of the carpus, the anterior row of which was very much exposed and loosened from its connexions with the other; the soft parts on the back, but particularly on the palm of the hand were extensively lacerated—hæmorrhage trifling."

The limb was removed a little above the middle of the fore-arm by the flap operation—some inflammation that subsequently occurred in the stump was subdued by a twelve-ounce bleeding from the arm, a purge, and the removal of the straps from the wound—the stump healed kindly—and the lad was discharged on the 27th of May, not quite three weeks from his admission.

Dr. Ballingall remarks that in a case which he saw of the circular operation in the fore-arm, the bellies of the muscles being full below the elbow, the skin, when divided by the first turn of the knife, could not be easily drawn upwards over the subjacent muscles, but formed on them a stricture, like that of the prepuce on the glans in phymosis. We do not consider this a serious objection, as its occurrence must be rare, and the tension of the skin might be readily removed by a trifling incision at the side of the limb. The flap operation in the fore-arm is a neat one, accomplished with ease, and subject on the whole to as few inconveniences as a flap operation can be. It occasionally, however, occurs that the tendons, "from the resistance they give to the knife, are in some measure drawn upwards, and are cut longer and less smoothly than they should be." The remark is a just one, for not only the tendons, but also the arteries and nerves are too often unequally cut. In the flap operation the incision is oblique, the division of the blood-vessels oblique, and hence we believe, it has resulted that the artery being injured above where the ligature was applied, the consequence

* Dr. Ballingall's Clinical Lecture, delivered to the Students of the Royal Infirmary, July, 1828.

has been the occurrence of secondary hæmorrhage. These circumstances of course, do not damn the operation, but merely teach us caution in performing it. Dr. Ballingall thinks that the simple expedient of making an assistant firmly grasp the wrist, will enable us to avoid the unequal division of the tendons.

The next case on the list is that of Alexander Moffat, ætatis 17, admitted on the 20th of May, with two contused wounds of the lower part of the thigh, the ham and the knee. The integuments and fascia were separated from each other all around the knee, and in many parts, the latter membrane was lacerated also. Two similar but slighter contusions existed on the ankle and heel. The injuries were caused by the wheel of a cart very heavily laden, pressing for some minutes against the limb.

"On the morning of the 21st, the wound was observed to be gangrenous, and before the usual hour of visit this gangrene had spread extensively round the knee and down the fore part of the leg; free incisions were made through the black and insensible skin, which gave vent to large quantities of fœtid air and dark coloured sanies. The propriety of amputating the limb was now considered in a full consultation, and was declined, in consequence of the advanced state of the disease, the lower part of the thigh being decidedly gangrenous, and the remainder of it so far involved as to be discoloured, swollen, tender, and emphysematous; the wounds on the other leg had also assumed a gangrenous disposition, the patient was affected with subsultus tendinum, his pulse at 120 and fluttering, his tongue furred and dry, his skin hot, and thirst urgent. Hot turpentine was poured into the incisions, and the effervescing poultice applied. Opium and wine were administered internally, but without any thing like even a temporary suspension of the symptoms. He soon became delirious, and expired on the evening of the 23d." 8.

As we purpose very shortly devoting an article to some cases of traumatic gangrene, we shall say very little on the subject at present. Our impression on perusing this case, is certainly in opposition to the determination that was come to by the gentlemen in consultation. The injury was received at the lower part of the thigh and the knee, and the gangrene at the hour of visit on the 21st, had only, we are told, "spread extensively *round the knee*, and down the fore part of the leg." If the operation is desirable at all in "traumatic gangrene," and Dr. Ballingall is one of those who think that it is, then surely it was needed in a case like the above. There may have been features in the case that induced the consultants to reject the operation, features of which we can possibly know nothing. Judging, however, from the litera scripta —the report, we certainly think that the thigh should have been amputated as near the trochanters as the surgeon could go. Whilst we state our own opinion, we think it only fair to let Dr. Ballingall speak for himself.

"Although, for the reasons formerly stated, I declined this operation in Moffat's case, and although the only case in which I have ever operated in such circumstances, terminated fatally, yet I should be sorry to have it thought that I am in any degree hostile to the practice. I think it right to observe, that, in declining an operation in the case now under review, I was in no degree influenced by the unfavourable issue of another case, which I shall immediately proceed to detail. You will recollect that at the time the amputation of Moffat's limb was under consideration, the case I allude to afforded a prospect of a favourable result. In declining the removal of this boy's limb, I was actuated by a conscientious conviction, (right or wrong,) of its inutility; by a firm persuasion that the performance of an operation in a case so hopeless, would have been more likely to bring a promising practice into disrepute, than to have saved the life of my patient."

The case that is alluded to above was under the care of Mr. Liston, who, it gives us much pleasure to find, is displaying his talents in the walls of the Royal Infirmary.

Robert Brockie, 40 years of age, was admitted, on the 3d of last May, having fallen from a house four stories high. The tibia and fibula were fractured, about an inch and a half above the ankle-joint, the lower portion of bone being forced beneath the upper. The second phalanx of the thumb was also broken. "The limb was placed on the suspending apparatus."

On the 7th, the bandages were removed from the ankle-joint, in consequence of pain and swelling of the limb. On the 8th, he was rather light-headed, and complained of pain of head. The pulse being full, he was bled to ℥xx. when the blood was both buffed and cupped. The skin around the fracture had a dusky red colour, and presented on the fore part of the leg some vesications—the pulse was 84—tongue loaded—bowels costive.

℞. *Tart. potass. et sod.* ℥ss. *Supertart. potass.* ℥ss. *Tart. antimon.* grs. ij. *Aquæ,* ℥xvi. ℳ. *Capt.* ℥j. *3tiâ quâq. horâ.*

On the ninth, the whole inside of the leg was discoloured, and a streak had extended up the thigh—there were several black vesications on the ankle—toes cold—much starting of the limb. A bandage was applied from the toes to the thigh,* and a lotion was used to the leg. He passed a bad night, undoing the bandages, and was certainly worse next day. *Infus. catechu thebai,* ℥ss. *subinde. Haust. s.* c. *Tinct. op.* gtt. *c.*

On the 11th, the appearance of the leg was much the same, but the fore part of the foot and the toes were livid and cold. Camphor, in almond emulsion, was given, with beef-tea ad libitum, and next day presented the following symptoms.

"Lies in a drowsy state, but frequently starts up in bed. The dusky appearance on inside of thigh has entirely disappeared, the fore-part of foot is more livid and cold; he does not appear to have any feeling in his toes, the discharge from the ankle has a most offensive smell; no stool since yesterday; tongue loaded; much thirst; his breathing appears rather laborious; delirious at times; no pain of head; pulse 100, of good strength; skin hot."

Mr. Liston now removed the limb, above the knee, by the flap operation, when the bones were discovered to be much comminuted, and the fracture extending into the ankle-joint. The cartilages of the latter joint were red, and very putrid matter was found to have burrowed in the calf of the leg. Some hæmorrhage followed the patient's removal to bed, in consequence of which the stump was undone, and several vessels were secured. We deem it unnecessary to go into the minutiæ of all the diurnal details; suffice it to say, that the patient next day was affected with troublesome cough, and, subsequently, pain in the breast. The pulse was about 80, and the bowels confined. On the 15th, bilious vomiting was added to the cough, the skin was cold and clammy, the pulse 55. On the 16th, hiccup supervened; the discharge from the stump was fetid and profuse; the bowels out of order. His nights were very restless—delirium came on—the hiccup was frequent—subsultus appeared on the morning of the 19th. The pain in the breast, on the 23d, was severe on a full inspiration, the cough very troublesome, and he died in the course of the 24th. The discharge from the stump appears to have been great, but latterly was healthier than at first, whilst nothing like a recurrence of the gangrene would seem to have taken place. The treatment consisted at first of camphor mixture; then of the tincture of digitalis and tincture of gentian; and, finally, he was supported by means of spirits—the camphorated tincture of opium—spiritus ammoniæ aromaticus, and camphor in almond emulsion, each being exhibited as the varying symptoms required.

Dissection of the body discovered a fracture of the 4th rib, an inch from the cartilage; a small amount of pus on the outside of the pleura costalis; and old adhesions of the pleuræ to a considerable extent. The left lung was full of those tubercular bodies that are found to be formed after great operations—there were several abscesses in the liver, and a quarter of a pint of bloody serum in the pericardium. A long coagulum existed in the femoral artery.

We agree with Dr. Ballingall, in thinking that the present case proves little in either way—against the operation during gangrene, or for it. We are free to confess, as they phrase it in Parliament, that we do not think the patient would have died one day sooner had the limb been left on; nor, perhaps, a day later had it never been touched. The cause of death was neither the operation nor its effects, but the purulent depôts in the liver and

* We do not see the reason for bandaging; in fact, we question the propriety of the measure.—Rev.

the lungs, dependent, in all probability, on the injury originally received. The local affection appears to have been mild, indeed we suspect that it would not of itself have proved fatal, had the viscera been free from disease. It seems to have been rather a form of erysipelatous inflammation than gangrene, or at least that acute and very dangerous variety that is frequently the sequel of a violent injury. We remember to have seen after simple fracture of the tibia, an affection of the leg very similar to this, consisting in numbness, discolouration, and black vesications, when the patient recovered without any operation or slough of more than the integument. In that case, the pulse running high, venesection was employed.

"One symptom, however, appeared early in this patient's case, which I did not fail to remark to my colleagues, and which, as far as my observation goes, is a circumstance almost uniformly foreboding a fatal termination; I allude to a peculiar yellow hue of the skin, which not unfrequently attends the symptomatic fever supervening upon wounds and operations; this has perhaps struck me more forcibly from being familiar with a similar appearance in the idiopathic fevers of tropical climates; and although I have no wish to alarm the citizens of Edinburgh by talking of a yellow fever in this part of the world, yet I am bound to state for your instruction, that I have occasionally seen it here as well marked as I ever saw it at Seringapatam or Batavia, and when supervening upon injuries, much more uniformly fatal.

"A case of this kind occurred some years ago, which made a deep impression on my mind, and which must have done so, I think, upon all those who had occasion to witness it; I allude to that of a seaman belonging to one of His Majesty's ships, in the roads, whose limb had been amputated below the knee in consequence of an accident. The accommodation on board his ship being defective, and the vessel about to sail, he was brought ashore to this Hospital and placed under my care; here his stump sloughed, the symptomatic fever ran high, was attended with that dingy yellowness of the skin to which I allude, and in a few days he died. I observed to the surgeon of the ship who came ashore to see him dissected, that this case wanted nothing but the 'black vomit' to constitute a complete example of yellow fever; and it was found on laying open the stomach, that this circumstance, necessary to complete the parallel, was hardly wanting; for here was a large collection of that dark grumous fluid resembling coffee grounds, which is so often evacuated from the stomach in tropical fevers."

We are somewhat at a loss to know whether Dr. Ballingall is alluding to a peculiar and uncommon appearance in disease, or one that we have repeatedly witnessed ourselves, and must have been as repeatedly witnessed by others. As far as we have seen, this yellow and bilious suffusion is generally noticed in cases of gangrene and phlebitis, a little before their termination. That at times it exists in other cases, we are very well aware, but we think it will be found a most constant attendant on these two diseases. The symptom has always been the harbinger of death, the constitution of the fluids appearing to be vitiated, the pulse being fluttering or feeble, the secretions from the wound being greatly perverted or totally arrested, and the powers of the mind subverted in delirium. Acknowledging, however, as we do, the fatality of the symptom, we cannot, at the same time, erect it into yellow fever with the facility of Dr. Ballingall. We should term it the precursor of death, not the cause; and consider it as marking the depravation of the fluids, the sequence of disease, rather than disease in itself.

Here we conclude for the present, with the intention of resuming the review of the Report on a future occasion. Before parting company, we beg to express our satisfaction again at the *course* Dr. Ballingall has taken.

---

### 79. La Pitie.

#### I. Amputation of the Neck of the Uterus.*

As operations on the uterus appear to be be growing very fashionable, perhaps it would be useful to give some account of the mode of proceeding adopted by Lisfranc.

---

* *La Clinique.*

The patient was a woman 49 years of age, and the operation performed on the 4th of July, in the following manner. The neck of the uterus being too much enlarged to allow of its being seized at the bottom of the vagina, it was necessary to draw it down to the orificium externum, in order to determine the limits of the disease. This preparatory step was, however, very difficult, in consequence of the tumour being fungous and softened, whilst there only existed a kind of band above, where the tissue of the organ was so firm as to admit of the necessary traction. This difficulty was surmounted, by M. Lisfranc introducing the fore-finger of the left hand so deeply, that its point passed the limits of the fungus, and then carrying forwards upon it a hook, (*une aizique*) guarded by lying with its flat side on the finger. Having reached the sound part of the uterus, the hook was turned round and impacted in its substance, by pressure with the end of the finger. Many other hooks were introduced in the same manner, and the diseased mass drawn down, by their means, to the labia externa, when a pair of forceps, (pince de museux) was applied, and several of the hooks were withdrawn. The tumour being retained in this position by assistants, the operator proceeded to ascertain the exact limits of the disease, and the point of attachment of the membrane of the vagina, rather by means of the finger than the eye. The labia were then separated as widely as possible, and the band of sound parts cut across by a bistoury with a very concave cutting edge, the section being made from behind towards the front, and care being taken to prevent any obliquity.

These were the principal features of the operation, which we give for the sake of recording the *how*, and not of discussing the *why*. The most difficult and embarrassing part of the proceeding, was getting the tumour to the mouth of the vagina, a difficulty that is sometimes completely insuperable. In a case of polypus uteri at St. George's, under the care of Mr. Brodie, the body was so large, that the efforts to draw it down were ineffectual, and in consequence abandoned. In a case which occurred to M. Dupuytren, the traction was also ineffectual, until the perinæum was divided! In the case of Mr. Brodie's to which we have alluded, the operation was concluded by throwing a ligature, by means of the double canula, round the neck of the polypus in situ. The ligature was tightened de die in diem, and the tumour sloughed off at the end of a week. The operation was accomplished in a very few minutes, occasioned but trifling pain, and produced no subsequent inconvenience whatever.

### II. Death from the Operation of making a New Nose.

A middle-aged man, of apparently good constitution, contracted the venereal, for which he was an inmate of the Hôpital des Vénériens, and treated by mercurial preparations. The malady was cured, but not before its ravages had entirely deprived him of his nose, in consequence of which he entered La Pitié, to undergo the operation for remodelling that feature on the "human face divine." M. Lisfranc, however, hesitated at first, from a fear that the system was not entirely purged of its syphilitic taint, or, as yet, in a proper condition for ensuring the surgeon's success. As Winter came on, the cold was supposed to be injurious; and early in the Spring, copper-coloured blotches appeared upon the surface, and delayed the performance of the long-expected operation until the 16th of last July.

The dimensions of the flap upon the forehead were not, as is usual, marked out by ink, but the nitrate of silver, in substance, to prevent their being effaced and washed away by the blood. The rule of not denuding the frontal bone so much as to cause its necrosis was attended to, and the incision on the left side was carried three lines lower than that on the right, in order to allow for the twisting of the flap. The operation being ended, the patient was taken to his bed, and the dressings not applied for an hour, to allow the bleeding from the smaller vessels to be stopped. The lips of the wound on the forehead were then brought together by adhesive straps, and covered with simple dressing, compresses, lint, and circular roller over all. The flap was applied upon lint covered with linen, arranged in the form of a nose, and supplying its place; its borders were connected with the groove by twelve or thirteen points of suture, and two adhesive straps crossed it transversely, the one at the top and the other at the bottom, surmounted by a piece of simple dressing and a bandage denominated a mask.

For the first few days after the performance of the operation, there was fever and want of sleep at nights. These unfavourable symptoms, however, passed away—the general condition of the patient promised well—and the artificial nose appears to have succeeded, when suddenly a change supervened, which rapidly hurried the patient to his grave. The report on the 28th, left the patient doing well, but he complained next day of a little diarrhœa; the tongue was dry, the abdomen not painful upon pressure. One or two whitish points which had previously appeared on the wound of the forehead, were now become black, but, other than these, no symptoms of importance, or, indeed, any symptoms all, are recorded. The patient expired at five o'clock that evening.

*Sectio Cadaveris* 40 *hours after death.*—The body was by no means meagre, or attenuated. The head and the chest shewed no signs of disease, except that, in the latter, there were traces of ancient adhesions of the pleuræ. The stomach shewed patches of red here and there—the duodenum bore marks of chronic inflammation, its mucous coat being thickened, and apparently puffy. Towards the termination of the small intestines, seven or eight ulcers were discovered, with thickening of the mucous membrane. The large intestine was the seat of acute inflammation—the spleen rather larger than usual—the liver was healthy and natural.—*Clinique.*

There is nothing, we think, in the foregoing dissection, which fairly accounts for the sudden termination, and the symptoms during life are even more ambiguous. We do not clearly see from the written report, which is certainly not quite so full as it might be, the immediate cause of the patient's disease. The reporter, however, has promised to clear up the mystery hereafter. The case, is very interesting, on several accounts, and calculated to teach those members of the profession who excel in this particular branch of their art, that they cannot always fashion new noses with impunity.

### 80. GLASGOW ROYAL INFIRMARY.

### HOSPITAL DISEASES.

We resume the review of Dr. Anderson's Clinical Report, by extracting some observations on what are termed by the Doctor "hospital diseases." After noticing the paucity of cases of erysipelas, he proceeds to observe—

"There were also fewer of the other *hospital diseases*, under which term I have been accustomed to include a great variety of inflammatory attacks, both external and internal; affecting sores, and remote from them. Many of the causes of these attacks are involved in considerable obscurity. I am inclined to attribute much to regulation of diet, cleanliness both personal and general, and ventilation. But there is another cause which I must not omit to notice. This is crowding, the direct operation of which, in generating many diseases, not generally attributed to hospital causes, does not in my observation admit of a doubt. This I have noticed amongst the medical patients; but it is still more conspicuous where there are a great number of sores discharging copiously. I have paid some attention to this subject, and I have refused to admit patients who required to be laid on additional beds on the floor. Whenever, also, there was any appearance of hospital disease, I have endeavoured to diminish the numbers as much as possible. The beneficial effects have corresponded very nearly with the practicability of effecting this and other objects connected with this important subject, into which I cannot here enter."

In the common acute erysipelas of the skin, superficial incisions were occasionally used, but the free use of leeches, with lotions, and salts with the tartrate of antimony internally were generally sufficient to check the disease. The erysipelas serpens of debilitated patients, or "travelling erysipelas,"* as Mr. Abernethy calls it, was treated, with success, by a dose or two of calomel and antimony, followed by the sulphate of quina and wine. When this form of erysipelas was circumscribed or confined to a limb, a narrow piece of blistering plaster placed

* Egad! it is a travelling disease.—*Abernethy's Lectures.*

on the surrounding sound skin was often sufficient to stop it.

The practice of blistering in the neighbourhood of erratic erysipelas we have never had an opportunity of witnessing, but local applications have generally so very little power in the disease, that we should not be disposed to place any great faith in the measure. The French apply the blisters on the surface immediately inflamed, a much more objectionable plan.

II. Exophthalmos—Successful Removal of the Eye.

Mrs. Craig, ætatis 24, was admitted into the Infirmary, Jan. 5th, with the following affection of the eye.. The right eye-ball was almost completely protruded from the orbit, attended with ectropion and chemosis; the cornea was ulcerated and muddy; the pupil immoveably dilated; the vision irrecoverably lost. Her health was impaired—she had constant severe pain in the bones of the orbit and right side of the head, increased upon pressure, and also some rheumatic pains in the knees. The vision of the eye had begun to be dim, about a year and a half before admission, and the sight was destroyed at the end of twelve months. The pain of the head had existed for a year—the prominence of the eye for eight weeks. At the time that the vision was lost, but not afterwards, she had some discharge of yellow fluid from the ear; her mouth was affected at the time of her admission, from the pills she had been taking for five or six weeks.

From the symptoms and history, Dr. Anderson suspected the existence of syphilis, but the patient denied it, and the mercury appeared to have no other effect than that of increasing the debility—opiates and narcotics were employed without effect—the humours were let out with the same ill success, and finally the organ was removed by the knife. A tumour, the size of a hazel nut, nodulated, solid, and bony, was discovered in the floor of the orbit, the pressure of its growth having obviously occasioned the protrusion of the eye. It was now firmly fixed, unable to exert any pressure, and, therefore, very prudently allowed to remain undisturbed.

Plummer's pill and decoction of sarsaparilla were employed for some weeks, at the end of which time she got almost well. The pain was remarkably relieved after the operation, and the patient left the house on the 1st of March, her health being good, and presenting no appearance of increase of the growth in the orbit.

III. Exostosis from the Dorsum Ilii.

John Henderson, ætatis 39, began in the early part of 1827, to suffer occasional pains about the left hip-joint and sacrum. These gradually increased, extending to the knee-joint and ankle, and after three months were so severe, especially in the groin and the inside of the thigh, as totally to prevent him from walking, whilst a swelling took place between the trochanter and the sacrum. Blistering, cupping, and caustic issue were employed without effect, and the patient was received in the Infirmary on the 8th of September. The left hip, though natural in shape, was greatly swelled, the swelling extending to the crest of the ilium, the median line of the sacrum, and half way down the thigh. There was no pain on pressing the articular surfaces together, or pressing the trochanter, but the patient was unable to rest on the limb from stiffness and pain. The thigh was bent forwards, and the limb seemed elongated half an inch, which was obviously owing to inclination of the pelvis. The health was giving way—the feet œdematous—the urine high coloured and scanty—the body emaciated.

The swelling of the hip and left side of the pelvis steadily increased, he became hectic, and affected with general anasarca, and died on the 22nd of November. Diuretics, mercurials, &c. were used—an incision was made just behind the trochanter to ascertain whether or not there existed any matter, and the question decided in the negative; and lastly, the hydriodate of potass was rubbed upon the hip.

*Dissection*. The hip was about double its natural size, the glutæi were wasted, and more superficial than the tumour. The bones of the left side of the pelvis, excepting the sacrum, were surrounded by a quantity of firm, slightly elastic, and semi-cartilaginous substance, which was readily cut, and shewed on its section a yellowish white appearance. On the dorsum of the ilium its thickness was from five to seven inches, on the internal surface of the pelvis it was not above four, but occupied the situations of the iliacus internus, levator ani, the psoæ, and obturator internus, the traces of which had entirely vanished, save, near the crest of the ilium, a very small portion of the iliacus internus. It also arose from the external margin of the foramen ovale, passed an inch or two down the femur, but did not adhere to its periosteum, and took an attachment to the anterior part of the os pubis, at which situation it was traversed by the femoral artery, unaltered in structure.

Those parts of the tumour that were near the periosteum, were traversed by spiculæ of new bone which was near an inch in thickness, behind the acetabulum. In all the other parts there was only a small quantity of new bone formed near the periosteum, but the tumour was firmer the nearer it was to the old bone, and firmer on the dorsum than inside of the pelvis. The hip-joint was unaffected—the thoracic and abdominal viscera were healthy.

This is certainly an interesting case, and presented many features in common with inflammation, or rather disease of the hip-joint. Dr. Anderson remarks, that it seems to have been the *fungous exostosis* described by Sir A. Cooper, in his Surgical Essays, but still does not think that like other exostoses, this would have been changed into bone, even had its growth and termination been slower.

"I believe it to have been originally a disease of the periosteum alone, and that the spiculæ of bone which were found, do not constitute an essential character of the complaint. I have in my possession a finger which I amputated, on account of a tumour of the same description with this, and although there are a few minute spiculæ at the point from which it took its origin, the whole tumour, *which was of sixteen year's standing*, and as large as a hen's egg, is still free of bony structure."

That the disease was a periostitis, or disease of the periosteum, there cannot exist much doubt, but we very much question from the spiculæ of bone, deposited in parts, whether that deposition would not have been greater, had the affection proceeded farther, or even more slowly. We could give a curious instance of bony and cartilaginous formations, if our limits permitted, but this not being the case, we must conclude.

---

### 81. HOPITAL ST. ANTOINE.

Cases of Pneumo-thorax. M. Rayer.

Of all the causes of pneumo-thorax, the most frequent is the opening of a pulmonary excavation into the cavity of the pleura. This accident is by no means so infrequent as some people imagine. Two cases have been witnessed in the above-mentioned hospital, in the course of a few months.

*Case* 1. *Pulmonary Tubercles—Perforation of the Left Lung—Pneumo-thorax—Considerable Displacement of the Heart.*

Sophy Gonce, aged 23 years, married, and the mother of one child, became affected with catarrh and palpitation of the heart some time previously, and stated that about six weeks before her entrance into hospital she felt something give way, while in the act of coughing, followed by great difficulty of breathing, [obliging her to remain in a particular position, to be noticed presently] palpitation in the *right* side of the chest, and some spitting of blood. On the 11th of March, the day after she entered the hospital, she presented the following phenomena:—The respiration, short and embarrassed, compelled the patient to remain, day and night on her knees and elbows in bed, the trunk inclining a little sometimes to the right, sometimes to the left. On percussion, the whole of the left side of the chest was extremely sonorous, while the right side sounded dull. The breathing was loudly heard in the upper part of the right side of the chest—no respiration in the left side. In the act of strong inspiration, however, a curious sound was heard through the stethoscope, resembling the blowing of air into a large bottle, (respiratio amphorica)—while, in gentle respiration, the tintement metallique (or sound of a drop of water falling into a bottle half full) was distinctly heard The pulsation of the heart was evident in the anterior part of the right side of the chest. M. Rayer wrote down in the case-book, as diagnosis—"*pulmonary phthisis—aperture of an excavation into the left pleura—communication of one of the bronchia with this cavity—heart in the right side.*" M. Rayer thought this last was congenital; but in this his diagnosis was wrong. The symptoms above-described continued, with little variation, till she died, on the 29th of March.

*Dissection.* The left side of the chest appeared more bulged out than the right, and the intercostal spaces wider. When the left side was opened, a strong gust of air rushed out—the lung was found to be almost annihilated, and the pleura covered every where with false membranes. An aperture was readily discovered in the lung, through which the air escaped into the cavity of the chest, when a pair of bellows was applied to the trachea. On more minute examination, a small excavation was found at this place, communicating with one of the bronchia. In the right side there was nothing particular. The heart had evidently been pushed over by the air in the left side of the thorax. There were tubercles in both lungs.

Case 2. *Pulmonary Tubercles—Perforation of the Left Lung—Pneumo-thorax—Displacement of the Heart.*

Lemaire, aged 24 years, married at the age of 15, and has borne six children. She entered the hospital on the 17th April, 1828. Stated that she had had some attacks of hæmoptysis the preceding year—and always brought up much expectoration, with very little cough. On examination, pectoriloquism was heard under the right clavicle—the respiration audible behind on both sides. Percussion did not elicit any thing particular. On the 25th April, the patient was found sitting on her bed breathing very laboriously, and complaining of an acute pain under her left breast. On examination by percussion, the left side of the thorax resounded very loudly—the respiration was extremely short, and incapable of being performed in the recumbent posture. When the ear was applied to that side, no respiratory sound could be heard, but only the "TINTEMENT METALLIQUE," as in the former case. Succussion was practised, and a fluctuation was distinctly heard in the chest. The right side was also sonorous, in a natural degree, and pectoriloquy was recognized under the right clavicle. The patient was occasionally seized with a paroxysm of coughing, during which she brought up a trifling expectoration, exhaling a most intolerable odour. The lower and anterior part of the right side sounded dull, on percussion, and here the pulsation of the heart was distinctly heard. The tongue was red—there was thirst—inclination to vomit—diarrhœa. *Diluents and abstinence.* Between the 25th and 30th the patient continued in nearly the same state, and she was visited by several physicians, among others M. Bricheteau, all of whom agreed that the disease was pneumo-thorax. The left side of the chest became more and more bulged out, and the intercostal spaces wider. Between the 1st and 4th of May, the symptoms were a little mitigated, and the patient walked about the ward a little every day. On the latter day, she was suddenly seized with an increase of the dyspnœa, and quickly expired.

*Dissection.* MM. Rayer, Kapeler, and Brichetcau, assisted at the examination. The right cavity of the chest was first opened, and there the heart was found to be located, being pushed over from the opposite side. The pleura of the left side was seen to be distended with air, and quite transparent. On being opened, a quantity of this fluid rushed out. The cavity contained a moderate quantity also of sero-purulent fluid, mixed with shreds of false membrane. The lung of that side was reduced to one-eighth of its natural dimensions, and compressed against the spine. The whole of the pleura was covered with false membrane, very thin in substance. A small aperture of communication between the air-tubes and the cavity of the chest was discovered in the contracted lung. In this channel of communication there was a small excavation, into which two or three bronchial tubes opened. Both lungs contained tubercles, and there was some bronchial inflammation.

*Remarks.* The foregoing cases are very interesting in themselves; but we think our readers can hardly fail to perceive that the accuracy of the diagnosis was not turned to any practical utility in the way of relief to the patients. Now we must acknowledge that the two individuals above-mentioned did not present, on dissection, any organic disease incompatible with life—or, at least, with a prolongation of life. They evidently died in consequence of the effusion of air into the cavity of the chest compressing the lungs, displacing the heart, and impeding the vital function of respiration. They were, in fact, placed as nearly as possible, in the same situation as individuals who had been wounded in the lungs by a small sword, or by the fractured ends of ribs. Why, then, hesitate to make an opening through the intercostal spaces, and extricate the effused air? Every time the patient breathed, some air was forced out into the cavity of the pleura, and it could not get back again through a valvular opening. The obvious remedy was a free exit from the chest, till the channel of communication closed. The operation could not have added to the danger of the patients, and it would have given them a temporary relief at least. We are the more surprised at the inactivity of the medical attendants, in the above cases, seeing that Laennec advises the operation "whenever there is imminent risk of suffocation." We shall introduce an extract from Laennec, touching the treatment of pneumo-thorax.

" *Treatment.* The exact diagnosis of pneumothorax, and of each particular variety of it, must not be considered as a matter of purely speculative knowledge, or as useful only in respect of the prognosis of the disease. It is extremely probable, as has been remarked by Hewson and Rullier, that simple pneumothorax is the case which holds out most prospect of success from the operation of puncturing the chest. This opinion is corroborated by an observation of Riolan, who informs us that he had several times seen the operation of paracentesis successfully performed on patients considered as affected with dropsy, but from whose chests only air made its escape. In cases of this kind, the puncture with the trocar would unquestionably be preferable to incision. But I would here remark, that, exclusively of the great infrequency of the simple pneumothorax, I think it must be generally considered as of no great severity, the gas being more readily absorbed than the liquid effusion. I think myself justified, at least, in drawing this conclusion from the frequency of gaseous effusions in other situations, which disappear spontaneously, and frequently in the course of a few days or even hours. Of this kind is the pneumo-pericardium, and the various kinds of *pneumarthrosis*, particularly that of the knee, which so frequently arises during the convalescence from articular rheumatism, as well as in other circumstances. On this account, before proceeding to puncture the chest, we ought to endeavour to excite absorption by aromatic and spirituous frictions and by the internal use of slight tonics. —Pneumothorax complicated with liquid effusion and still more with pulmonary fistula, is a case of a most serious nature, and leaves little hopes of a cure being effected. This, however, must not be considered as quite impossible, even in the severest cases. I formerly proved the possibility of the cicatrization of tuberculous excavations; and the observations of Bacqua, Jaymes, and Robin, (Journ. Gen. de Med. 1813), to which I could add a more recent case of the same kind (I mean cases where the patients recovered after the operation of empyema, although the injections thrown into the wound were found to be discharged by the mouth) sufficiently prove, that, even in such cases, we may attempt this last resource with some prospect of success. Even Nature by herself may sometimes overcome more or less completely, a lesion of the kind in question, as I shall show in a case to be detailed at the end of the present chapter. I saw another case of the same kind in 1820, in a man who came on horseback thirty leagues, to consult me. In this person there was every sign of the complication in question existing on the right side. The disease was of two years standing, and nature had already made considerable progress towards a cure, as the affected side was evidently contracted. I ascertained in 1824 that this man was still alive, and attending to his business; he was improved in health, though still an invalid. It cannot be denied, however, that cases of this kind are exceptions to the general rule; and that the two last varieties of pneumothorax afford much less chance of success from the operation of empyema, than the simple effusion, whether of air or liquid. Accordingly, I think that we ought never to attempt this operation in such cases, *unless there is imminent risk of suffocation, or rapidly increasing emaciation and debility;* and never after the long continuance of the disease, unless the lung on the sound side, gives no indication of tubercles. In every other case, I think that we ought to content ourselves with supporting the patient's strength, promoting absorption by the means formerly mentioned, and by a regimen regulated according to the state of the digestive functions,—neither too rigid nor too analeptic."

II. Abdominal Fœtation.—M. Rayer.

C. Marie Jeane, aged 73, a widow, who had borne children, entered the hospital above-mentioned on the 27th February, 1828, presenting the following symptoms :—several parts of the surface were covered with eruptions of different kinds ; one was prurigo—another appeared to be of a *syphilitic* character, although the mouth, throat, and genital organs were free from trace of syphilis. After being treated a certain time for the cutaneous affections, the patient began to complain of head-ache, bad taste in her mouth, anorexia, and pain in the epigastrium. Leeches to the epigastric region. Erysipelas now broke out on the face, and required very active antiphlogistic treatment before it was subdued. A relapse took place, and the old lady sunk under symptoms denoting affection of the brain.

*Dissection.* The meninges were injected—there was ramollissement in the left hemisphere of the brain—thoracic viscera sound—108 biliary calculi in the gall-bladder. While examining the small intestines a tumour was discovered in the mesentery, containing a small fœtus, doubled up, and about two inches in length, with some parts imperfectly developed—others entirely wanting. There was nothing particular to be seen in the uterus or urinary apparatus.—*Archives.*

---

### 82. ROYAL WESTMINSTER OPHTHALMIC HOSPITAL.

Mr. Guthrie has been engaged during the last year and a half in ascertaining the efficacy of various remedies in curing the different forms of acute and chronic inflammation of the eye, and in making comparative trials of each. This has been done not only for the improvement of science generally, but for the purpose of obtaining better methods than those usually pursued for the relief of the more obstinate forms of disease. We think he has succeeded in many points, and it is now our intention to lay several of the cases before the public, in which the new method of treatment has been adopted. We have often heard Mr. Guthrie remark that remedies rarely did good in chronic affections of the eye unless they gave pain, and that even amaurotic cases were not always exceptions to the rule. The excitement of a different action to that already existing in the part, is the principle on which he has acted, and the remedies have been various:—the potassa fusa, argentum nitratum, cupr. sulphas, hydrarg. oxymurias, spirit. rosmorin, spirit. camphoræ, &c. They have been either used pure or combined in different ways. Mr. Guthrie prefers ointments composed of the argentum nitratum, and the hydrargyr. oxymurias. The argentum nitratum has been used from one to 15 grains, reduced to an impalpable powder, carefully mixed with one drachm of adeps. At present, from two to ten grains are made into an ointment with ʒj. of ung. cetacei. and 15 drops of the liq. plumbi subacet. The oxymuriate ointment is used in the proportion of two to four grains mixed in a similar manner. Mr. Guthrie gives the preference on the whole to the argentum nitratum ointment, called by the patients, from its turning black, the black ointment, at the strength of 10 grains to a drachm of the white ointment, and 15 drops of the lead. These ointments all give pain ; in some persons it is considerable, lasting from one to 12 hours according to the length of time the ointment has been made, and the peculiar irritability of the patient. The newer the preparation, the more severe in its effects, the decomposition which takes place not being completely accomplished. It has been used in all kinds of disease, chronic, acute, catarrhal, and purulent inflammations, in iritis corneitis, amaurosis. It has cured cases of all of these complaints, and many which have resisted other means of relief. The re-application of the ointment rarely takes place before the third day; sometimes it is repeated on the second, and occasionally only on the sixth, seventh, or eighth. When the effect of them seems stationary, Mr. Guthrie frequently alternates their use with other remedies, such as the sulphate of copper, warm water, blisters, purgatives and cupping.

*Case* 1. Maurice Bryan, aged 46, has been suffering from sore eyes for the last nine years, and has been frequently unable to get his bread, although he has been under several eminent medical gentlemen and in hospitals for relief, in vain. June 17, came to Mr. Guthrie; the conjunctivæ of the lids were thickened and irregular on their surface, and very red, the eyeball vascular, the corneæ muddy, sight very imperfect. The ung. argent. nitrat. was immediately applied to both, six grains of calomel with colocynth at night, and an ounce of sulph. of magnesia in the morning. 21st. The same repeated —absented himself until the 8th July, when recourse was again had to the ointment, the eyes being better. 10th. Ung. argt. nitrat. pil. hydr. subm. gr. vj. mag. sulph. ℥j. 15th. Ung. argt. nitrat. 17th. Rep. ung. et pil. 19th. Ung. 21st. Ung. argt. nitrat. et pil. 24th. Declares he is much better than he has been for years. 26th, 29th, 31st. Aug. 2nd, 5th. Improvement continues, and says he is wonderfully well, and shall attend from time to time.

*Case* 2. Eliza Edwards, aged 18, had had bad eyes for several years, and attended different gentlemen, as well as at this Infirmary, for several months, at various times, with little relief. In the beginning of January last, began the use of the ung. argent. nitrat. which she has continued until the middle of June, with different intervals, taking nothing but aperient medicines occasionally. The relief obtained was such, that in May she thought herself nearly cured, and her father returned thanks to Mr. Guthrie; but she has remained till now, the 17th June, in order to secure her from relapse.

*Case* 3. Thomas Walsh, admitted March 3, having had sore eyes for more than five years, and has been under the care of several gentlemen during that period. The conjunctivæ are thickened and granulated—the corneæ opaque and vascular—he sees very badly. The ung. argent. nitr. to be applied every visit, with aperients. 10th April. The inflammatory appearances of the eyeball, as well as the muddiness, nearly removed. He declares the application has acted like a charm, and that the left eye is cured. Aug. 12th. The ointment has been continued twice a week to the right eye, which he says has some little remaining the matter with it.

*Case* 4. William Wood has had sore eyes for more than two years and a half—was attacked by inflammation of the eyes, which was very severe. After a time, he applied to Mr. Guthrie; but not getting well, he was induced to leave him, and go to another gentleman, who took him into an hospital for a month, and made his mouth sore. He then became an out-patient, and remained under his care for six months longer, but without any advantage. His disease having lasted now 2 years, for the last eleven months of which he was unable to see to get his bread, or do any thing; he returned to Mr. Guthrie, Jan. 3d, 1828. The ung. argent. nitrat. was applied to both eyes, and repeated on the 6th and 10th, 15th and 20th. On this day, he declared he could see to do many things he had not been able to do for a year, and that he was very much better. The ointment was repeated 23d, 27th, 31st, Feb. 5th, 7th. 19th. Absented himself from the 7th to the 19th, having been able to go to his work, which he is now able to do. 21st, 26th. Ointment applied. March 10th. Says he now goes to work regularly, but will attend from time to time.

April, May, June. Comes occasionally, and has the ointment applied to one eye; the eyeball is free from disease—the eyelids have still some chronic inflammation and redness affecting them, but he is careless about it, as it does not interfere with his pursuits. He has only taken a purgative occasionally.

*Case* 5. Emma Dagnie, aged 10, 12th June. Catarrhal inflammation of both eyes. On the left cornea there is a small ulcer: the complaint has lasted six weeks, and came on after the measles. Hydr. submur. gr. iv. h. s. Magnes. sulph. ℥ss. mane. Ung. argent. nitrat. to both eyes. 14th. Much better. repeat the ointment.

17th. Nearly well—repeat.
19th. Well.

*Case* 6. John Davis, aged 45, admitted July 4, 1828; pustular inflammation, which commenced suddenly last Tuesday morning with a sharp, shooting, pricking pain, as if something had got into the eye. There is a great discharge of hot tears—no pain in the fore-head, but a sensation of fulness and intolerance of light. The ung. argent. nitrat. applied to both eyes. Pil. hydr. subm. gr. vj. statim—magnes. sulph. ℥j. omni mane.
5th. Better, pil. hydr. c. gambogia, gr. x. h. s.
6th. Ung. argent. nitrat. cum pilulis, hydr. c. gambog.
8th. Ung. argent. nitrat. *Cured.*

*Case* 7. Eliz. Lewis, 26th July, 1828, inflammation and thickening of the semilunar fold of the conjunctiva of the right eye, extending inwards—pain and discharge of tears—intolerance of light slight—no internal medicine. Ung. arg. nitrat. 29th. Better—rep. 31st. rep. Aug. 5. *Cured.*

*Case* 8. Nicholas Metrelin, aged 49, July 31, catarrhal inflammation severe and acute, and of seven days standing. The ung. argent. nitrat. to the right eye. The ung. oxymur. hydr. to the left. Aug. 3. Says the ointments have done him a great deal of good, and is very thankful for the relief. Repeat the ointment. 5th. Is nearly well. 7th. Discharged cured—no internal treatment.

*Case* 9. Henry Clifton, aged 14, August 5, 1828, catarrhal inflammation slight. Ung. argent. nitrat. August 7. Cured by one application.

*Case* 10. Edward Dudley, aged 5, Aug. 5, 1828, pustular inflammation. Ung. argent. nitrat. Pulv. jalap. c. ʒss. 7th. Cured.

---

### 83. HOPITAL SAINT LOUIS.

### I. Eczéma Rubrum.

Antoine Massonot, ætatis 26, of strong and sanguineous temperament consulted M. Biett in March, 1825, in the following condition. The hair, which had recently been cut, was soaked in an abundant ichorous discharge; the scalp was very red, and enveloped, as it were, in a whitish and shining kind of cap, resembling asbestus in appearance; this cap came away in fine scales, beneath which appeared innumerable vesicles. The ears and meatus externus were covered also with small white scales, and the trunk and axillæ were likewise affected. The patient, when young, had twice had the itch, of which he was perfectly cured. At the age of 18, he was subject to epistaxis, and at 22, after leading an irregular life, an eruption appeared upon the face, and excrescences on the gland of the penis, for which he was put under several mercurial courses. In the Winter of 1824, the eruption disappeared, but as the cold weather went off, the combination of symptoms, for which he consulted M. Biett in the month of March, was gradually established. The patient had made trial of the mineral waters of Barége without advantage, and now, after seeing M. B. determined on entering the hospital. The digestion was good, but M. Biett, notwithstanding, resolved on employing the purgative plan of Dr. Hamilton, under the influence of which the little scales fell off, the kind of cap upon the scalp became loosened, and moisture re-appeared in abundance.

A gap intervenes in the report until January of the present year, when the state of the patient appears to have greatly improved. There merely existed on the scalp, a few small lenticular scales, which were readily detached; but the itching continued unabated. The patient, perceiving the excellent effects of the purgation, had taken clandestinely much greater doses than were ordered, and at last paid the penalty in the shape of disturbance of the bowels, and diarrhœa. These symptoms were checked by pediluvia, infusion of mallows in emulsion, and pills of the pulvis lactucæ and extract of hemlock, but during their use two large red patches, without any vesicles appeared upon the temples. In April, the patient was ordered the vapour bath, and frictions with ioduret of sulphur. On the 24th of May, the bowels having resumed a healthy condition, he began to rub in upon the tongue, 2,6me and afterwards 3,6me of muriate of gold. Under this treatment, the disease disappeared—the hairs became natural, and healthy at their roots—the scaly eruption vanished from the scalp—the itching subsided, and the patient was discharged on the 24th of July. The excrescence on the gland had been snipped off with scissors, and subsequently cauterised with the nitrate of silver.

The eczéma rubrum, according to M. Biett, generally occupies the head, seizing on the scalp and the face. It begins with an eruption of extensive and numerous vesicles, accompanied with a bright erythematous red. The vesicles soon give way to squamæ with a constant discharge, the itching is great, the ears and meatus auditorius very frequently affected, the scalp is enveloped in an "anianthus-like" cap, which has caused M. Alibert to give it the name of TINEA ANIANTACEA.

II. ELEPHANTIASIS ARABUM.

Two patients affected with this melancholy and disgusting affection are at present inmates of Saint Louis. The one is an old man, sixty-five years of age, who has spent a great part of his life in the Antilles, the other a child born in Saint Domingo, and not having been above eighteen months in France. In the former the disease was much farther advanced than in the latter. Its ravages are confined to the head and face, the trunk of the body being totally exempt. The tongue is affected with tubercles, which have even gone on to ulceration in the palate; the pharynx and larynx are apparently affected, the voice being hoarse. The iris is irregular and deformed on the right side, but almost entirely destroyed on the left. The vision on this side is lost of course; on the other it is very much weakened. In the child the disease is not near so extensive, the palate and tongue being sound, but the voice is rendered hoarse notwithstanding. This hoarseness of the voice the reporter considers a very common symptom, and constantly present in the cases of leprosy, which exist in great numbers in the neighbourhood of Marseilles, and the villages round about Martignes. The treatment adopted in the cases the subject of report, is a vegetable diet, the exhibition internally of the tincture of iodine, in doses of 15 or 20 drops, and frictions with the ioduret of sulphur, rubbed upon the tubercles. The young patient promises fair for recovery, the old one posseses a very indifferent chance of it.

Some interesting clinical remarks on the disease were made by M. Biett at the bedside of the patients. This physician has never seen a case of true elephantiasis Arabum in France, unless the individual had either been born between the Tropics, or dwelt there some time. The reporter, however, believes from the cases he has witnessed, that the disease may be developed in France, and especially the south. With regard to diagnosis; the yellow colour of the skin, from which the tubercles arise—the peculiar appearances presented by the latter, separate at first, but afterwards agglomerated together, indurated, irregular, and insensible—the partial death of the parts which are attacked—the peculiar aspect—and hoarseness of the voice, are symptoms which cannot be mistaken. The tubercles may ulcerate, but generally the matter is pent up within them, when it forms, and is not discharged for a great length of time. The eruption is mostly confined to the limbs and the face, and seldom seizes on the trunk.

M. Biett has seen the tubercular deposits in the alimentary canal, especially in the neighbourhood of the ileo-cæcal valve. He has also seen ulceration of the arytænoid cartilages and chordæ vocales—the liver enlarged and tuberculated—the bones sometimes altered and softened, their cancellated structure inflamed. The ulceration which takes place at the summit of the tubercles on the arch of the palate, penetrates by a sort of infundibulum, into the interior of the mucous follicles, a circumstance which leads M. Biett to believe, that it is in these that the tubercles originate. The skin over the tubercles in the cutis or sub-cutaneous cellular membrane, is sometimes so attenuated, as to look like the finest parchment.

We pass to the treatment of this curious and interesting malady. Mercury is by no means a favourite with M. Biett, indeed he believes that in one instance, that of an old lady, it induced the formation of tubercles in the lungs. Arsenical preparations are said to be useful, the Indians employing the protoxide in combination with pepper. M. Fodéré praises the remedy highly, and the "pilulæ asiaticæ" appear to have occasionally succeeded, though attended with very bad consequences in a case which was recently received in the Saint Louis. In one case M. Biett employed the muriate of gold in frictions on the tongue, with partial good effects. The tincture of iodine, with frictions of the ioduret of sulphur has removed the tubercular deposits from the limbs, and restored sensibility to the parts and power to the legs. At the commencement of the disease, before the development of the tubercles, rubefacients and blisters to the part are of service.

---

### 84. WORCESTER INFIRMARY.

MISPLACED COLON, WITH ENTERITIS.

Ann B. was a patient in the Worcester Infirmary in the course of the year 1821, with a fixed tumour in the right iliac region, painful and tender. The bowels were costive—abdomen tense—tongue foul—pulse quick and weak—emaciation considerable. Under the employment of a seton in the situation of the tumour, low diet, and occasional doses of castor oil, the tumour very nearly disappeared, the other symptoms yielded, and she gradually recovered.

After a violent paroxysm of anger in October, 1823, she was suddenly attacked with a fixed and very violent pain in the bowels of the "true inflammatory character." The seat of the former disease was the most tender part of the abdomen—the bowels, at first loose, became afterwards obstinately bound, and she vomited bilious matter. The treatment consisted in local and general bleeding, in spite of which the abdomen, in 72 hours from the seizure, became tympanitic—the pulse was too quick to be counted, and the patient soon afterwards expired.

*Sectio Cadaveris.* The omentum, at its lower edge, was inflamed, and adherent in the right iliac region to the peritoneal lining of the abdominal muscles, as well as to the subjacent intestines. A portion of the omentum was sphacelated. The transverse arch of the colon, instead of traversing the epigastric region, descended from the right hypochondriac to the right iliac, and then again ascended to the left hypochondrium, from which it continued its natural course. The peritoneal coat of the centre of the arch had contracted the principal adhesions with the omentum, and was thickened to nearly a quarter of an inch, over a space about as large as three half-crowns. Part of the peritoneal coat of the colon was gangrenous, but the villous was healthy, and throughout the whole tract of the intestines. The diseased part of the colon was adherent to the cæcum below, but the latter was free from disease. Lymph was thrown out on the folds of the intestines, and there existed a pint of serum in the cavity of the peritoneum.

In the following remarks we agree with the reporter.

"The diseased portion of the intestine, which evidently constituted the original tumour, was, from its situation, considered by all who saw her when she was in the Infirmary to be the cæcum. The fact of the displacement of the transverse arch, could not be ascertained in the life-time of the patient. This case shews how rapid and severe is acute inflammatory action, when it takes place in a morbidly altered structure, in which, perhaps, chronic inflammation is going on. It shews, too, that those violent passions of the mind which augment generally the force and rapidity of arterial action, are powerful exciting causes of local inflammation."—*Midland Reporter, No.* 1.

---

### 85. HOTEL DIEU.

PERFORATION OF THE ORIGIN OF THE DUODENUM FROM ULCERATION OF THAT INTESTINE—SUPERACUTE PERITONITIS. By M. ROBERT.

*Case.* Louis Laurin, aged 17 years, had complained for some months of fugitive pains in the epigastrium. For the last six weeks there had been diarrhœa, and for ten days past there was loss of appetite, nausea, and general malaise. On the 10th of December last, three hours after dinner, he was seized suddenly with most violent pains in the region of the stomach, which radiated thence over the whole abdomen. He vomited up what he had eaten mixed with some bile and mucus. A physician who was called in, considered the case as one of indigestion, and prescribed chamomile tea and a lavement. Next morning the patient was carried to the HÔTEL DIEU. His face was pallid, skin covered with cold perspiration, pulse small and quick, abdomen tense and very painful, tongue pale and moist, bilious vomiting. Forty leeches to the abdomen. The patient died at 4 o'clock the same afternoon.

*Dissection.* The abdomen was filled with a gazeous and fetid fluid—peritoneum inflamed in streaks—intestinal convolutions agglutinated together. There was, in some points of the intestinal tube, a veritable sub-peritoneal emphysema. The mucous membrane of the stomach appeared sound. At the origin of the duodenum, immediately beyond the pyloric orifice of the stomach, there was an oval ulceration, three or four lines in diameter, with smooth and somewhat raised edges. In the centre of its bottom the peritoneal coat was perforated by a small opening, a line in diameter, thus affording a free communication with the cavity of the abdomen. Near this ulceration a second was discovered, not quite so large, and not penetrating deeper than the mucous membrane. The rest of the intestinal canal was perfectly sound.

It is very rare to find the duodenum perforated by the ulcerative process, the stomach and intestinal canal remaining in a healthy condition.—*Biblioth. Med. Juillet.*

---

### 86. ST. GEORGE'S HOSPITAL.

#### Erysipelas.

The following is a highly interesting case.

Joseph Smith, ætatis 53, was admitted into hospital, under the care of Mr. Brodie, on the 27th of August, in consequence of extensive varicose ulcers of the right thigh and leg. The limb had been affected in a similar manner twenty years ago, but the ulcers at that time were perfectly healed, and broke out no more till within three months, or so, previous to admission. The treatment which was ordered was—quietude in bed—bandages—and strapping. These means were, accordingly, adopted, and the plaister renewed about every other day. On the 3d of September, the limb was strapped as usual, and the dressings were about to be removed on the 5th, when the thigh and the leg were found to be enormously swollen, and the seat of extremely severe erysipelas. The colour of the inflammation was dull and opaque, but not absolutely dusky—the pulse weak and rapid—the tongue rather foul, but not brown—the surface much heated—the countenance sallow and marked with anxiety. The bowels were confined, but the patient had very little thirst, and no head-ache whatever. On inquiry it was found that on the preceding evening he complained of some nausea, without any shivering or actual sickness. He suffered much pain that night and next morning, on account of the tightness of the bandage, which caused, as has been stated, much swelling above and below. Violent applications had been used by the dresser to take down some high granulations, but these were desisted from several days previous to the 5th.

*Cal.* gr. iij. *Pulv. Jacob.* gr. iij. *statim. haust. sennæ post* 6 *horas—Lot. frigid. membro affecto.*

In the evening, the pulse was 150 and tremulous; tongue white and furred; skin burning hot. The bowels had acted three times since the morning, and the patient declared he was better. On the 6th, there appeared some improvement. The pulse had got down from 150 to 126, the skin was not so hot, the tongue a little cleaner, the erysipelas fading in parts of the thigh. On the next day, however, the features of debility were more unequivocal, and verging a little to the typhoid character. The tongue was now dry—the pulse had again risen to 140—the animal powers seemed low and weak.

℞. *Liq. ammon. acetat.* ℥ss. *Ammon. carb.* gr. v. *Mucilag. acacia,* ʒij. *Syrup.* ʒj. *Aq. distill.* ℥v. ℳ. *6tis horis.*

When we saw him on the 8th, his appearance was most unpromising. The hue of the countenance sallow before, had now become sallower still, anxious, and half-hippocratic; the tongue was glazed with brown, and as dry as a board in the centre, but red at the edges and tip—the pulse very rapid and weak—skin hot—manner very hurried and delirious. Suspecting the existence of thoracic or abdominal inflammation, we examined the patient attentively, but discovered no symptom of either. The erysipelas was fading, but dusky; no particular pain on pressure of the thigh or over the femoral vessels.

*Haust. cinchon.—tinct. opii* ♏. iij. M. *6tis horis.*

On the 9th, he appeared to be sinking, the surface having assumed a yellow, bilious hue—the erysipelas spreading up the trunk, and having terminated in vesications on the outside of the thigh—the tongue being brown—mind wandering—subsultus. He died this evening.

On dissection, it was found that the knee-joint contained four ounces of pus, and its cartilages were extensively ulcerated. The blood-vessels were healthy, but serum and lymph existed, and that to some extent, in the cavity of the pleuræ.

The above case was curious in many respects. The sudden, and almost universal appearance of inflammation on the limb, foretold, but too truly, how severe, or even fatal the attack would be. When we saw him on the 8th, the general character of the symptoms, the extraordinary depression, the delirium, not loud nor loquacious, but rather a quickness of manner, and eager desire to convince you he was well, the sallow, we should say, icteritious hue of countenance, and lastly the deep and inexpressible anxiety depicted in the features, induced us to suspect the formation of matter in the chest, the abdomen, or vessels of the thigh. In this, as it happened, we were partly deceived, but the purulent depôt in the knee-joint, and destruction of its cartilage, without even a symptom, or shadow of a symptom, during life, is analogous in principle, with the other more frequent affection. The whole is instructive in the highest degree.

---

### BIBLIOGRAPHICAL RECORD.

1. Deafness; its causes, prevention, and cure. By John Stephenson, Esq. M.R.C S. Lecturer on the Structure, Economy, and Diseases of the Eye and Ear, &c. &c. 8vo. pp. 262. Colburn's, London, 1828.

2. The Constitution of Man, considered in relation to external objects. By George Combe. 12mo. pp. 319. Edinburgh and London, 1828.

3. A View of the Elementary Principles of Education, founded on the Study of the Nature of Man. By G. Spurzheim, M.D., &c. 8vo. pp. 327. June, 1828.

4. On Difficult Cases of Parturition; and the Use of Ergot of Rye. By W. Michell, Member of the Royal College of Surgeons. 8vo. pp. 128. London, 1828.

5. A Letter to the Right Honourable Robert Peel, on the supply of Water to the Metropolis. By Robert Masters Kerrison, M.D. 12mo. pp. 34. stitched, London, 1828.

6. Lexicon Pharmaceuticum, or a Pharmaceutical Dictionary, comprehending the Pharmacopœias of London, Edinburgh, and Dublin, &c. &c. Designed for the use of Students. By Thomas Castle, F.L.S., M.R.C.S., &c. Second edition, with corrections, and an additional Appendix of the new Remedies. 18mo. pp. 354. Cox, London, 1828. price 9*s*.

7. Synoptical Table of Midwifery, shewing the management of Natural and Difficult Labours—their Consequences and Treatment. By Henry Hurry Goodeve, and Thomas Evans, late House-Pupils to Dr. E. J. Hopkins, &c.—Highley, Fleet Street.

8. The Morbid Anatomy of the Bowels, Liver, and Stomach, illustrated by a series of Plates, from Drawings after Nature; with explanatory letter-press, and a Summary of the Symptoms of the Acute and Chronic Affections of the above-named Organs. By John Armstrong, M.D Lecturer, &c. &c.

Fasciculus 1. pp. 48. (4to.) with fi[illegible] plates.

Fasciculus 2. pp. 22. (do.) with five plates. Baldwin, & Co., July 1828.

9. Two Lectures on the Application of the Stethoscope to the diagnosis and treatment of Thoracic Diseases. By William Stokes, M.D., Physician to the Meath Hospital. 8vo. pp. 54.

10. Second Statement by the Council of the University of London, explanatory of the Plan of Instruction. 8vo. pp. 168. London, 1828.

11. A Series of Observations on Strictures of the Urethra; with an Account of a new Method of Treatment, successfully adopted in Cases of the most obstinate and aggravated Form of that Disease; illustrated by Cases and a Plate. By Richard Anthony Stafford, M.R.C.S., &c. 8vo. pp. 159. London, 1828.

12. A Medico-Legal Essay on Infanticide, translated from the Author's Latin Inaugural Dissertation. By Robert Arrowsmith, M.D., President of the Royal
